# Extraction, Phytochemical Analysis, and Bioactivity Evaluation of Polyphenols from *Kunzea ericoides* (Kanuka) Plant

**DOI:** 10.3390/antiox14111319

**Published:** 2025-10-31

**Authors:** Harmandeep Dhaliwal, Yan Li, Michelle Yoo

**Affiliations:** 1 School of Science, Auckland University of Technology, Auckland 1010, New Zealand; harmandeep.dhaliwal@autuni.ac.nz (H.D.); yan.li@aut.ac.nz (Y.L.); 2 Maurice Wilkins Centre, Auckland 1010, New Zealand

**Keywords:** kanuka, total phenolic content, antiproliferative activity, antioxidant activity, triple-negative breast cancer, LC-MS

## Abstract

*Kunzea ericoides* (kanuka), a native plant of New Zealand, has a significant role in traditional medicine due to the presence of essential oils. Apart from these oils, this plant also is a source of many bioactive compounds, majority of which are polyphenols. However, there is lack of sufficient data supporting the extraction of polyphenols from kanuka plant leaves and investigating its bioactivity and phytochemical properties. The study aims to extract polyphenols from kanuka plant leaves with a conventional solvent-based method and determine the phytochemical analysis as well as bioactive potential. Extraction was performed with methanol and acetone as solvents. Polyphenolic prolife was analyzed with LC-MS. Bioactive analysis of kanuka leaf extract was carried out to determine total phenolic content and antioxidant activity. We investigated the cytotoxic effect of kanuka leaf extract on two triple-negative breast cancer cells—MDA-MB-231 and BT-549. LC-MS analysis confirmed kanuka leaf extract is a source of many polyphenols, some giving very prominent signals on TIC scan. Ten polyphenolic compounds were confirmed to be present in kanuka leaf extract based on MRM analysis. FRAP-CUPRAC analysis indicated significant antioxidant activity in the kanuka leaf extract. Antiproliferative analysis has confirmed cytotoxicity of the kanuka leaf extract on the triple-negative breast cancer cell lines. This study indicates that *Kunzea ericoides* leaf extract, rich in polyphenols, shows promising antioxidant and antiproliferative potential, warranting further investigation for therapeutic applications.

## 1. Introduction

*Kunzea ericoides*, commonly called kanuka, is a native New Zealand plant that belongs to the *Myrtaceae* family. It plays a vital role in traditional Māori medicine because of known phytochemical and therapeutic properties. Studies confirmed that the essential oil of kanuka contains alpha-pinene and 1,8-cineole, which have strong anti-inflammatory properties [1]. The terpenes present in kanuka have antibacterial and antifungal activity, making it useful in skin conditions like acne, eczema, fungal infection and wound healing [2]. The safety and efficacy of 3% topical kanuka oil cream was investigated on eczema and a significant improvement in symptoms was reported [3]. A study analyzed the effect of kanuka oil on different strains of bacterial and fungal growth as well as lower tumor necrosis factor-α in THP-1 cells, confirming antimicrobial as well as an anti-inflammatory role of kanuka oil [4]. Kanuka oil has also been traditionally used in aromatherapy as the volatile terpenes promotes mind relaxation and steam inhalation of kanuka leaves ease respiratory discomfort [5]. Amongst various plant parts (stem, flowers, roots, bark and fruits), leaves are identified as a rich and accessible source of bioactive compounds [6]. Due to the role of leaves in photosynthesis and defense, numerous studies have confirmed that leaves have a higher accumulation of polyphenolics like flavonoids and hydroxycinnamic acids in comparison to stems or roots. Studies have confirmed the highest antioxidant activity in leaf extracts, correlating with high flavonoid content in multiple New Zealand medicinal plants [7,8]. Analysis of the foliage chemistry of ten Kunzea species from different geographical regions in NZ confirmed the presence of mono- and sesquiterpenes, flavanones, and low amounts of triketones in all samples. This study also determined that the chemical analysis of the Kunzea species foliage was not different based on the geography of the sample [9]. A recent study has evaluated volatile organic compounds present in kanuka leaves using subcritical water extraction in comparison to the conventional methods including steam distillation and ethanol-based extraction. Data showed composition of oil extracted from kanuka plant leaves containing terpenes, alkanes, esters, ketones and alkenes [10]. Although kanuka is traditionally known for a range of benefits due to its essential oils [11], interest has grown in modern research regarding the polyphenolic content present in this plant. Polyphenols are the secondary metabolites present mainly in the leaves and bark of plants and exhibit antioxidant, anti-inflammatory and anticancerous properties. A study identified chlorogenic acid, gallic acid, quercetin, and (E)-ferulic acid as the major bioactive compounds in kanuka leaf extract. These potent antioxidants help reduce oxidative stress, suggesting that the extract can delay skin aging by enhancing moisturization, providing UV protection in HaCaT cells, and inhibiting melanogenesis in B16F10 cells [12]. Investigation of the selective recovery of phenolic and flavonoid from kanuka leaves using subcritical water extraction and conventional ethanol-based extraction at different extraction temperatures, times and dry weight-to-solvent ratios confirmed the presence of eight compounds in the extract as well as a direct correlation between the total phenolic content and antioxidant activity of the samples [13]. Among various extraction techniques, conventional methods have remained fundamental in both scientific research and industrial settings due to their ease of use, affordability, and effectiveness, particularly when working with polyphenol-rich plant parts like leaves [14]. Maceration, which involves immersing finely ground or powdered plant material in an appropriate solvent, is one of the oldest extraction practices, and is extensively utilized for isolating heat-sensitive phytochemicals. This technique allows polyphenols to diffuse into the solvent without applying high temperatures, thus preserving their integrity [6]. For polyphenol extraction, aqueous methanol in concentrations between 50 and 80% is particularly favored for its ability to extract both water-soluble and slightly lipophilic constituents [15]. Given the thermal sensitivity and oxidative susceptibility of many polyphenols, maceration is especially beneficial, offering a gentle extraction environment that maintains the structural integrity and bioactivity of these compounds.

Recent advancements in LC–MS methodologies have greatly enhanced the sensitivity, resolution, and structural annotation of plant-derived phenolics. A study utilized an HPLC–QTOF–MS approach for comprehensive phenolic profiling [16], while another study [17] successfully isolated flavonoid glycosides from *Canavalia gladiata* pods using chromatographic separation and LC–MS. These works highlight the potential of advanced LC–MS techniques to deepen understanding of complex polyphenolic mixtures. In contrast, the present study employs a conventional LC–MS system to provide foundational evidence of the polyphenolic composition of *Kunzea ericoides* leaves and to establish baseline data for future application of these advanced analytical methods. Recent progress in LC–MS has refined polyphenol profiling across various plant matrices and studies available on kanuka plant have emphasized on antioxidant potential of essential oils only; studies on polyphenols present in kanuka plant leaves remain limited. Therefore, this study offers a comparative evaluation of methanolic and acetone-based maceration extracts of kanuka leaves, correlating their phenolic content and antioxidant activity with cytotoxic effects on TNBC cell lines. By integrating chemical characterization with functional bioassays, this work presents a novel perspective on the therapeutic potential of this Māori plant beyond its well-known essential oil composition and provides a framework for future LC–MS-based investigations. The study aims to extract polyphenolics from *K. ericoides* leaves collected in the spring season (September) from plants grown in the Gisborne region of New Zealand. Two maceration-based extraction methods were compared: (i) acetone containing 1 mg/L tetrabromophenyl porphyrin (TBP) and (ii) 50% methanol diluted with Milli-Q water. The total phenolic content (TPC) and antioxidant activity of each extract were determined using the FRAP–CUPRAC assay, and the polyphenolic profile was analyzed by LC–MS. Finally, the cytotoxic potential of kanuka leaf extracts was evaluated against two TNBC cell lines, MDA-MB-231 and BT-549, to explore their potential anticancer properties.

## 2. Materials and Methods

### 2.1. Equipment

Lyophilization of the kanuka plant leaves was acquired by freeze dryer (CHRIST LOC 1 m, Martin Christ, Osterode am Harz, Germany). Concentrate of leaf extract was prepared by vacuum concentrator (Concentrator plus, Eppendorf, Auckland, New Zealand). UV–spectrophotometer (Genesys 150-thermo scientific, Auckland, New Zealand) was used to record absorbance of the samples after biochemical analysis. LC-MS analysis was conducted using an Agilent 1260 Infinity Quaternary LC System (Agilent Technologies Inc., Santa Clara, CA 95051, USA). Tecan Spark^®^ Multimode Microplate Reader, (Tecan Group Ltd., Männedorf, Switzerland) was used to measure absorbance of samples from 96-well plate.

### 2.2. Chemicals and Reagents

The chemicals and reagents used for the analysis were AnalaR grade or better. Dimethyl sulfoxide (DMSO), gallic acid, Folin–Ciocalteu reagent, neocuporine, copper (II) chloride, 2,4,6-tris(2-pyridyl)-s-triazine (TPTZ), Trolox (6-hydroxy-2,5,7,8-tetramethylchromane-2-carboxylic acid), and sodium carbonate were purchased from Sigma-Aldrich, Auckland, New Zealand. Methanol and acetone were obtained from Thermo Fisher Scientific, Auckland, New Zealand. MTT [3-(4,5-dimethylthiazol-2-yl)-2,5-diphenyltetrazolium bromide] was supplied by Sigma-Aldrich (St. Louis, MO, USA).

Analytical-grade standards of phenolic compounds—namely gallic acid, p-coumaric acid, epicatechin, caffeic acid, catechin, quinic acid, and quercetin were also procured from Sigma-Aldrich (Auckland, New Zealand). Additional reference standards including kaempferol rutinoside, chlorogenic acid, and pinocembrin were obtained from Phyproff^®^ Reference Substance, Vestenbergsgreuth, Germany.

Stock solutions of all pure phenolic compounds were prepared in DMSO at a concentration of 1 mg/mL and stored at –20 °C. Working solutions were freshly prepared in methanol by serial dilution, starting from 50 µg/mL of each stock solution to generate eight serial concentrations.

### 2.3. Plant Material

Kanuka plant leaves were collected in early spring (September month) from the plants grown in Gisborne, New Zealand. The plant material was thankfully gifted by Mr Alex Radley.

### 2.4. Cell Culture and Maintenance

The human breast cancer cell lines, MDA-MB-231 and BT-549, were procured from the American Type Culture Collection (ATCC, Manassas, VA, USA). Cell lines were cultured in complete medium prepared by Dulbecco’s Modified Eagle Medium (DMEM supplemented with 10% (*v*/*v*) FBS and 2 mmol/L L-glutamine in a humidified atmosphere of 5% CO_2_ at 37 °C. These cell lines were maintained for further research by splitting them 24–48 h periodically.

### 2.5. Lyophilization/Freeze-Drying

Leaves were ground into powder form using a pestle and mortar to reduce the size of the particles and increase surface area of contact between the sample and the solvent used for extraction. Collected powder was stored at −20 °C. For lyophilization, freeze dryer was pre-cooled to −80 °C and the samples of frozen kanuka leaf powder were freeze-dried for 48 h. Care was taken to begin freeze drying as soon as possible with the frozen samples to avoid thawing. As the freeze-drying ended, the samples were immediately stored at −20 °C to prevent rehydration.

### 2.6. Maceration-Based Extraction of Polyphenols from Kanuka Leaf Powder

Maceration is an extraction procedure in which the plant part is soaked in the solvent for a specific period. To extract polyphenols from the kanuka leaves, two maceration-based solvent extraction methods were employed following the method described by Diep et al. [18] with some modifications. The standard volume and concentration of solvents for maceration-based extraction as described by Diep et al. was followed to carry out the plant extraction.

#### 2.6.1. Method 1: Using Acetone with 1% Tetrabromophenyl Porphyrin (TBP) as Solvent

To a mass of 50 mg of freeze-dried and powdered kanuka leaves, 1 mL of 70% acetone containing 1 mg/L tetrabromophenyl porphyrin (TBP) was added. After brief vortex, the mixture was incubated in dark at 4 °C for 5 min and centrifuged at 4000 RCF for 5 min at 4 °C. The pellet was discarded, and supernatant was placed in an amber 1.8 mL glass vial and stored at −20 °C until further analysis.

#### 2.6.2. Method 2: Using Methanol as Solvent

The extraction of polyphenols from Kanuka plant leaf powder was carried out using 50% methanol. A total of 50 mg of freeze-dried powdered kanuka leaf sample was mixed with 1 mL of 50% methanol. The mixture was homogenized and incubated in the dark at 4 °C for 30 min, with vortexing for 30 s every 5–10 min. Following incubation, the samples were centrifuged at 10,000 RCF for 10 min at 4 °C to induce phase separation. The resulting supernatant was collected into a fresh tube and concentrated using a vacuum concentrator set to concentrator mode with solvent type alcohol (V-AL). The concentrated extracts were then transferred into 1.8 mL amber glass vials and stored at –20 °C until analysis.

### 2.7. Total Phenolic Content (TPC) Analysis

To compare the phenolic content in samples obtained by using methanol and acetone as solvents, total phenolic content identification assay was carried following the method of Dorman [19] with some modifications.

#### 2.7.1. Standard Preparation

Gallic acid, a phenolic compound, was used to prepare a reference standard curve. A gallic acid stock solution (1 mg/mL) was prepared by dissolving 10 mg of gallic acid crystals in 10 mL of distilled water. From this stock, five working concentrations (50–800 mg/L) were obtained by serial dilution using Milli-Q water.

#### 2.7.2. Sample Preparation and Analysis

A total of 0.1 g of freeze-dried kanuka leaf powder was placed in a 15 mL centrifuge tube and mixed with 4 mL of 50% methanol. The sample mixture was vortexed, homogenized, and left to stand at room temperature for 1 h. It was then centrifuged at 1500 rpm for 15 min, and the supernatant was transferred into a 10 mL volumetric flask. In a separate 15 mL centrifuge tube, 0.1 g of powdered kanuka leaf sample was extracted with 4 mL of 70% acetone, followed by homogenization, incubation (1 h at room temperature), and centrifugation under the same conditions. The resulting supernatant was collected in another 10 mL volumetric flask. Both flasks were then made up to volume with deionized water. From each extract, 1 mL was transferred into microcentrifuge tubes for further analysis.

For calibration, 1 mL aliquots from each of the five gallic acid dilutions (50–800 mg/L) and 1 mL of sample extracts were placed in separate microcentrifuge tubes. To each tube, 500 µL of Folin–Ciocalteu reagent was added, vortexed for 30 s, and incubated in the dark at room temperature for 5 min. Subsequently, 750 µL of 10% sodium carbonate (Na_2_CO_3_) was added, and the tubes were kept covered in the dark for 30 min.

Absorbance was measured at 765 nm using a UV–vis spectrophotometer (Genesys 150, Thermo Fisher Scientific) against a blank prepared with distilled water following the same procedure. Results were quantified using a gallic acid calibration curve and expressed as milligrams of gallic acid equivalents per gram of dry weight (mg GAE/g DW).

### 2.8. Antioxidant Activity of Kanuka Leaf Extract

#### 2.8.1. Ferric Reducing Ability of Plasma (FRAP) Assay

The FRAP assay was performed based on the method described by Benzie and Strain [20], with minor modifications. Kanuka leaf extracts were prepared using 50% methanol and 70% acetone. The FRAP reagent was freshly prepared in a 1:1:10 (*v*/*v*/*v*) ratio by mixing 20 mM ferric chloride, 10 mM TPTZ (prepared in 40 mM hydrochloric acid), and 300 mM acetate buffer (pH 3.6). For the assay, 1 mL of kanuka leaf extract was diluted with 9 mL of Milli-Q water to serve as the sample solution. A total of 2 mL of FRAP reagent was then added to 1 mL of the diluted sample. The blank was prepared by replacing the extract with Milli-Q water.

Both sample and blank solutions were vortexed for 30 s and incubated at room temperature for 5 min. Absorbance was measured at 593 nm using a UV–visible spectrophotometer (Genesys 150, Thermo Fisher Scientific). Antioxidant capacity was determined using a Trolox calibration curve (5–160 mg/L) and expressed as micromoles of Trolox equivalent antioxidant capacity per gram of dry weight (µmol TEAC/g DW).

#### 2.8.2. CUPric Reducing Antioxidant Capacity (CUPRAC) Assay

The CUPRAC assay was carried out following the method of Özyürek [21] with slight modifications. The prepared extracts were diluted 100-fold with Milli-Q water prior to analysis. For each assay, 1 mL of the diluted extract was combined with 1 mL of 0.01 M cupric chloride, 1 mL of 1.0 M ammonium acetate buffer (pH 7.0), and 1 mL of 0.0075 M neocuproine in 96% ethanol, resulting in a final reaction volume of 4 mL. The mixtures were incubated at room temperature for 5 min, after which absorbance was measured at 450 nm against a Milli-Q water blank using a UV–visible spectrophotometer (Genesys 150, Thermo Fisher Scientific). Antioxidant activity was quantified using a Trolox calibration curve (10–160 mg/L) and expressed as micromoles of Trolox equivalents per gram of dry weight (µmol TEAC/g DW).

### 2.9. Cytotoxicity Analysis of Crude Kanuka Plant Extract Obtained Using 50% Methanol Extraction

The effect of kanuka leaf extracts on the viability of TNBC cells, MDA-MB-231 and BT-549, was evaluated using MTT-based assay in 96-well plates. Cells were seeded in microwell plates at a density of 20,000 cells per well and cultured at 37 °C in a humidified atmosphere. After 24 h of incubation, eight concentrations of kanuka leaf extract (0.5–0.004 mg/mL) were prepared by serial dilution in complete medium from a 50 mg/mL filtered crude extract and applied to the wells. Following an additional 24 h incubation, 12 mM MTT solution was added to each well and incubated for 3–4 h. Formazan crystals were dissolved by adding DMSO to all wells (blank, control, and treatment). Absorbance was recorded at 540 nm with 680 nm as the reference wavelength using a Tecan Spark^®^ Multimode Microplate Reader (Switzerland). Cytotoxicity was determined relative to the untreated control and expressed as percentage cell viability.

### 2.10. LC-MS Analysis of Kanuka Leaf Extract

LC–MS analysis was performed using an Agilent 1260 Infinity Quaternary LC System (Agilent Technologies, Santa Clara, CA, USA) consisting of a 1260 quaternary pump (model G1311B), 1260 Infinity ALS sampler (model G1329B), 1260 Infinity TCC column compartment (model G1316A), and a 1260 Infinity diode array detector (DAD, model G4212B), coupled to a 6420 triple quadrupole LC-MS system equipped with an electrospray ionization (ESI) source (model G1948B).

Chromatographic separation was carried out on a Phenomenex Kinetex Evo C18 column (Phenomenex NZ, Auckland, New Zealand) (2.1mm × 150 mm). The mobile phases consisted of water with 0.1% (*v*/*v*) formic acid (solvent A) and acetonitrile with 0.1% (*v*/*v*) formic acid (solvent B). The flow rate was maintained at 0.30 mL/min. The gradient program was as follows: 95:5 (A:B) held for 0.5 min, increased to 15% B from 0.5 to 2 min, to 20% B from 2 to 9 min, and to 50% B from 9 to 11 min (held for 2 min). From 13 to 14 min, B was further increased to 80% (held for 2 min), then decreased to 5% from 16 to 17 min. The total run time was 27 min.

Compounds were identified and quantified based on their retention times and mass spectra compared with authentic reference standards. Preliminary results indicated that the retention time of the observed peaks was consistent with the presence of various phenolic compounds. Multiple Reaction Monitoring (MRM) was employed for the detection and quantification of these analytes. The MS ionization source conditions were as follows: capillary voltage of 4 kV, cell accelerator voltage of 7, drying gas temperature of 300 °C, drying gas flow of 10 L/min, nebulizer pressure of 40 psi. Positive and negative scans with a scan range from 100 to 1000 Da were performed for phenolic compound identification in kanuka leaf extract.

### 2.11. Statistical Analysis

All the experiments were performed in triplicates to obtain independent measurements, based on which mean and standard deviation were calculated. A *p* value of <0.05 was considered statistically significant. Pearson’s correlation coefficient was carried out to determine correlation among total phenolic content and antioxidant assay using FRAP and CUPRAC assays. Whole data analysis was performed using GraphPad PRISM8 (GraphPad; Dotmatics, Boston, MA, USA) software.

## 3. Results

### 3.1. Total Phenolic Content (TPC) in Kanuka Leaf Extract

The data for TPC analysis was presented as mg gallic acid equivalent per gram of dry weight. Using the linear equation y= mx + b, concentration of phenolics was calculated in different samples obtained by treating kanuka leaf powder with 50% methanol and 70% acetone. This concentration was equivalent to the mg gallic acid equivalent/L. calculated total phenolic content in the samples in GAE/mg of dry weight using equation TPC = (c × v)/m, where c is the concentration obtained from the standard curve using linear equation, v is the volume of the extract, and m is the mass of powdered leaf sample in grams.

The TPC of the sample extracted from dried and powdered kanuka leaves using 50% methanol was found to be 1035.34 mg gallic acid equivalents (GAE) per g of dry weight, with a standard deviation of 10.53. In comparison, the TPC of the extract obtained using 70% acetone was 886.91 mg GAE/g dry weight, with a standard deviation of 16.70. These results indicate that the methanolic extract contains higher total phenolic content than the acetone-based kanuka leaf extract.

### 3.2. Antioxidant Activity of Kanuka Leaf Extract by FRAP and CUPRAC Method

#### 3.2.1. FRAP Assay

Using the linear equation y= mx + b from the Trolox standard curve, µmol Trolox equivalent concentration per liter for each sample was calculated. Trolox equivalent antioxidant activity of each sample was calculated as µmol TEAC per gram of dry weight of the sample using equation (x × v × dilution factor)/dry weight, where x is the concentration obtained from the standard curve using linear equation, v is the total volume of reaction mixture (3 mL), dilution factor was 10 and dry weight of sample was 0.1 g.

The antioxidant activity of kanuka leaf extracts prepared using 50% methanol and 70% acetone was evaluated and expressed in µmol TEAC per gram of dry weight. The extract obtained with 70% acetone exhibited an antioxidant activity of 202 µmol TEAC/g DW, with a standard deviation of 8.915. In contrast, the extract prepared with 50% methanol showed a significantly higher antioxidant activity of 1054 µmol TEAC/g DW, with a standard deviation of 19.22. This indicates that the methanolic extract possessed approximately five times higher antioxidant capacity than the acetone-based extract.

#### 3.2.2. CUPRAC Assay

Using the linear equation y= mx + b from the Trolox standard curve, calculated µmol Trolox equivalent concentration per liter for each sample and using equation (x × v × dilution factor)/dry weight, the antioxidant activity was calculated as µmol TEAC/g DW. For CUPRAC assay, volume of the reaction mixture was 4 mL, dilution factor was 100 times and the dry weight of kanuka leaf powder was 0.1 g.

The antioxidant activity of kanuka leaf extracts prepared using 50% methanol and 70% acetone was evaluated and expressed in µmol TEAC per gram of dry weight. The extract obtained with 70% acetone exhibited an antioxidant activity of 573.74 µmol TEAC/g with standard deviation of 34.47 and the extract prepared with 50% methanol showed antioxidant activity of 446 µmol TEAC/g DW with standard deviation of 22.32.

**Figure 1:**
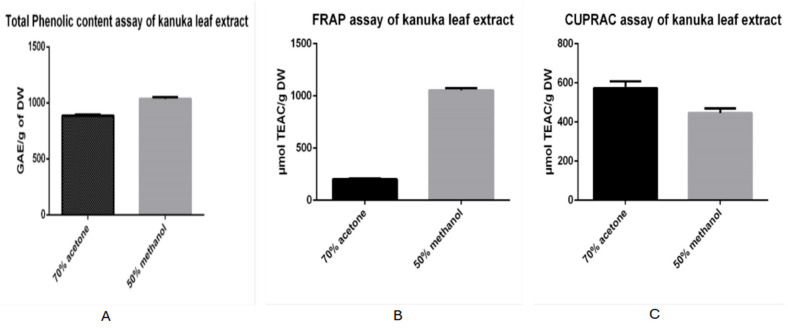
Histograms comparing total phenolic content (**A**) and antioxidant activity; FRAP (**B**) and CUPRAC (**C**) assay of methanol (grey bar) and acetone-based (black bar) kanuka leaf extracts.

Figure 1 shows the data of TPC, FRAP and CUPRAC assay results obtained for both 70% acetone and 50% methanol-based extracts expressed in histograms.

### 3.3. Correlation Between Total Phenolic Content (TPC) and Antioxidant Activity (CUPRAC and FRAP)

Results from Pearson’s correlation coefficient revealed a strong correlation between total phenolic content and antioxidant activity of the kanuka leaf extract obtained by the 50% methanol-based solvent method (Table 1), showing a linear relationship between both parameters. With a correlation coefficient of 0.845, 50% methanol-based kanuka leaf extract showed a statistically significant positive correlation (TPC-CUPRAC).

### 3.4. Cytotoxicity Analysis of Crude Kanuka Leaf Extract on MDA-MB-231 and BT-549 Cell Lines

**Figure 2:**
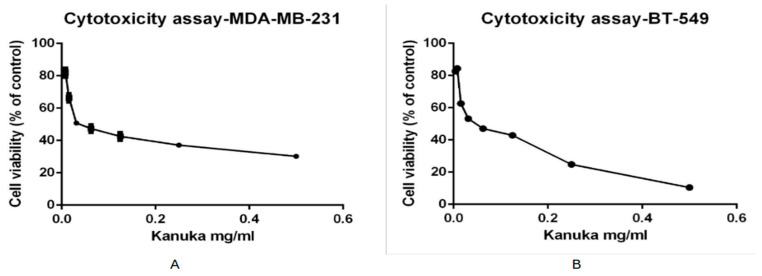
Cytotoxic effect of different dilutions of kanuka leaf extract (extracted by 50% methanolic extraction method) on MDA-MB-231 cells (**A**) and BT-549 cells (**B**) with seeding density of 20,000 cells incubated for 24 hrs.

As per the results from the TPC analysis of kanuka leaf powder, the 50% methanol-based extraction has a higher level of total polyphenolic content. To determine the cytotoxic activity of kanuka leaf extract obtained by using 50% methanol, eight different dilutions of the plant extract were prepared in complete medium. Triple-negative breast cancer cell line, MDA-MB-231 and BT-549 cell lines, were incubated for 24 h with each dilution of kanuka extract and tetrazolium dye was added to determine the cytotoxic effect of kanuka leaf extract on the cancer cell line. Figure 2 shows the cytotoxic effect of kanuka leaf extract on MDA-MB-231 and BT-549 cells after 24 h incubation with different serial dilutions of kanuka leaf extract.

The viability of the MDA-MB-231 cell line increased with a decrease in the concentration of kanuka leaf extract. A 50% and higher viability of the TNBC cells was observed above 1:1600 dilution of kanuka extract. The standard deviation values across all samples ranged from 0.599 to 2.368, with an average SD of 1.521. This indicates moderate variability across different replicates. A similar effect of kanuka leaf extract was seen with the BT-549 cell viability. The standard deviation values ranged from 0.831 to 1.849, with a mean of 1.353. The lowest cell viability was observed at 1:100 dilution of kanuka leaf extract, which was 30.12% (SD = 1.024) for the MDA-MB-231 cell line and 10.50% (SD = 1.42) for the BT-549 cell line, respectively.

### 3.5. LC-MS Analysis of Kanuka Leaf Extract

The kanuka leaf extract obtained by 50% methanol-based method was further tested to determine the polyphenolic profile using LC-MS.

#### 3.5.1. Total Ion Chromatogram Scan

**Figure 3:**
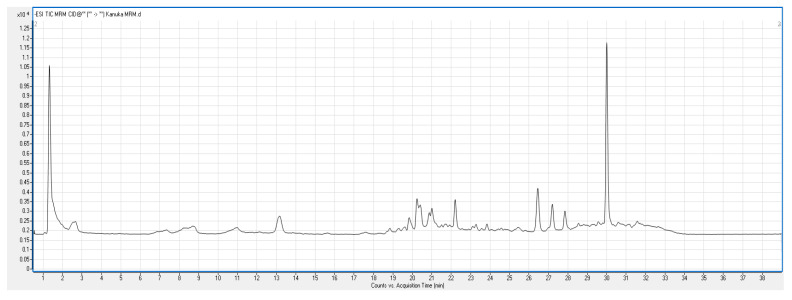
Positive TIC scan of phenolics in kanuka leaf extract with three prominent polyphenolics and corresponding peaks.

Total Ion Chromatogram (TIC) scan of kanuka leaf extract was carried out in both negative and positive ionization modes. With this scan, individual phenolic compounds present in kanuka extract obtained by 50% methanolic extraction were identified. Figure 3 shows the positive TIC scan of kanuka leaf extract with peaks and retention time for different polyphenols present in the extract.

**Figure 4:**
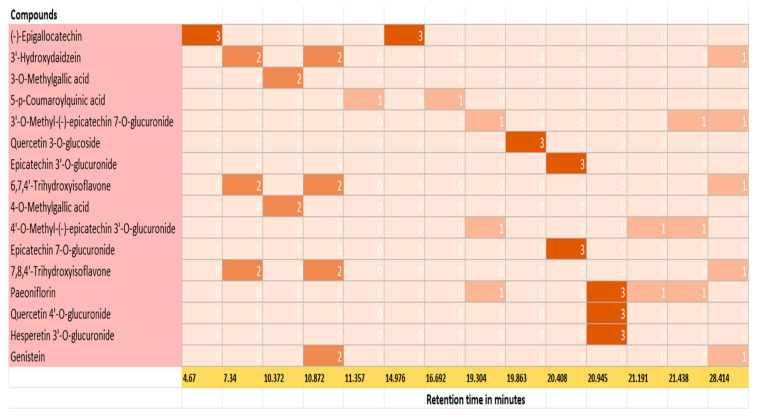
Heat map for polyphenolic compounds identified in 50% Me-OH based kanuka leaf extract based on the peaks and retention time in TIC scan; 0 represents no signal and 3 represents a prominent signal strength corresponding to each compound. Epigallocatechin, Quercetin 3-O-glucoside, Epicatechin 3′-O-glucuronide, Quercetin 4′-O-glucuronide, and Hesperetin 3′-O-glucuronide showed a prominent signal on TIC scan which corresponds to value 3 on the heat map.

The individual polyphenol compounds present in the extract were identified using the database of Phenol Explorer. The potential phenolic compounds based on the signal strength present in the kanuka leaf extract are listed in Table 2; using this data, a heat map was generated (Figure 4) separating tentative compounds based on the signal intensity generated in TIC scan.

#### 3.5.2. Multiple Reaction Monitoring (MRM) Validation for Major Phenolic Compounds

**Figure 5:**
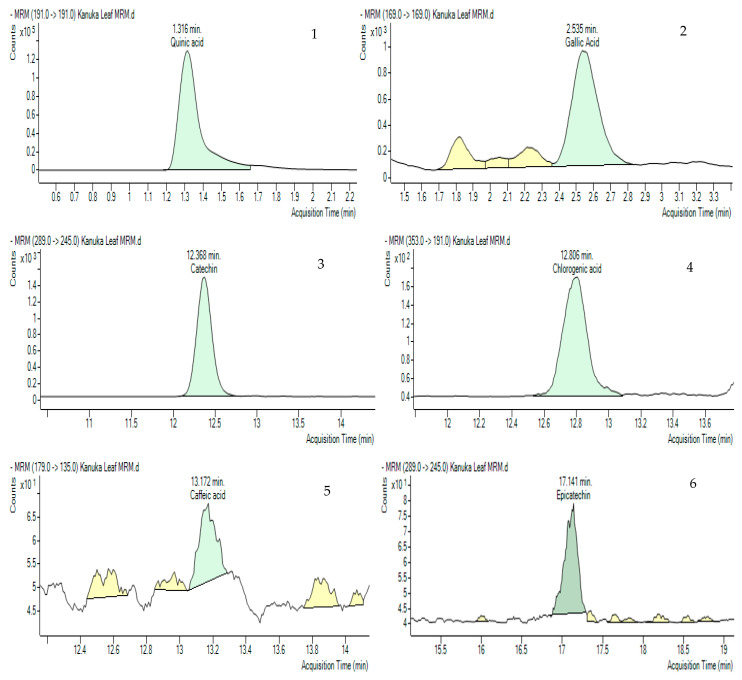

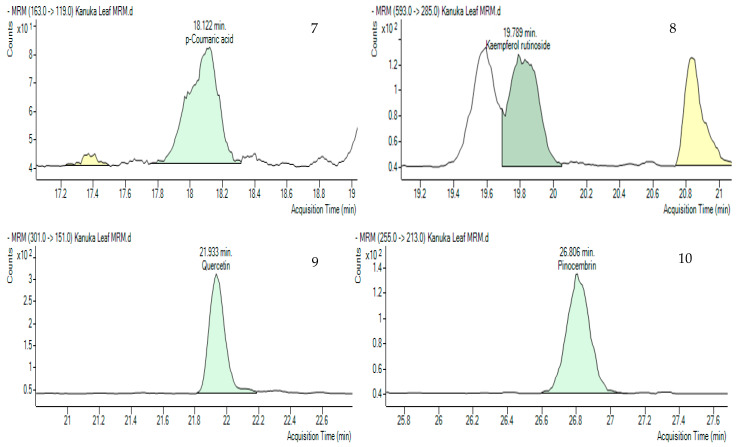
Multiple Reaction Monitoring (MRM) chromatograms (green-colored peaks) of 10 polyphenols in 50% Me-OH based kanuka leaf extract with the acquisition times. 1. Quinic acid (1.316 min), 2. Gallic acid (2.535 min), 3. Catechin (12.368 min), 4. Chlorogenic acid (12.806 min), 5. Caffeic acid (13.172 min), 6. Epicatechin (17.141 min), 7. p-Coumaric acid (18.122 min), 8. Kaempferol rutinoside (19.789 min), 9. Quercetin (21.933 min), 10. Pinocembrin (26.806).

**Figure 6:**
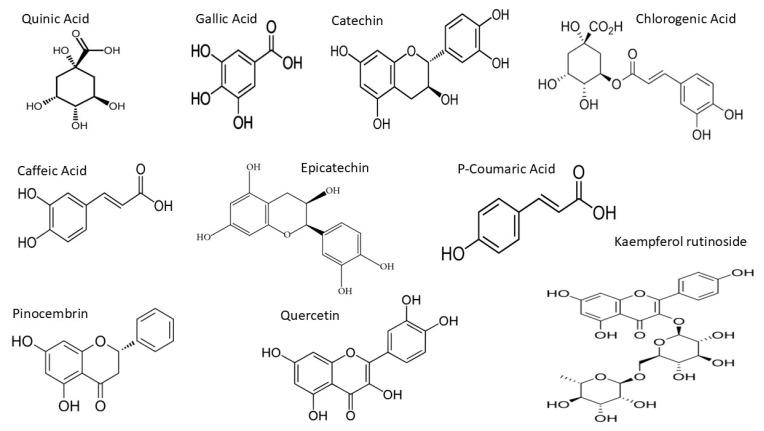
Chemical structures of the compounds identified in MRM of kanuka leaf extract.

Multiple Reaction Monitoring (MRM) was employed to detect and quantify polyphenols in the kanuka leaf extract. The optimized MRM transitions for the target compounds—including retention time, precursor ion (*m*/*z*), product ion (*m*/*z*), and collision energy (V)—are summarized in the Methodology section. Representative chromatograms of standard injections are shown in Figure 5 and chemical structures of the compounds identified in MRM are shown in Figure 6.

## 4. Discussion

The findings of this study demonstrate that solvent polarity plays a crucial role in determining the extraction efficiency, antioxidant capacity, and cytotoxic potential of Kanuka (*Kunzea ericoides*) leaf extracts. The 50% methanolic extract exhibited a significantly higher total phenolic content (TPC) than the 70% acetone extract. This result can be attributed to methanol’s higher polarity and its capacity to form hydrogen bonds with the hydroxyl groups of phenolic compounds, which enhances solubilization of polar phenolic acids and flavonoid glycosides. In contrast, acetone, being less polar, favors the extraction of moderately nonpolar compounds and consequently yields a lower phenolic content. Similar solvent-dependent differences in polyphenol extraction efficiency from *kanuka* and related species have been reported in previous studies employing subcritical water or mixed-solvent systems [13].

The antioxidant assays further revealed solvent-specific patterns of activity. The methanolic extract showed greater activity in the FRAP assay, implying that methanol extracts more water-soluble and fast-reacting antioxidants that perform effectively under acidic conditions. A positive linear relationship was observed between TPC and antioxidant capacity, indicating that phenolic constituents are the principal contributors to the antioxidant potential of kanuka leaf extracts. Comparable associations between phenolic content and antioxidant activity have been documented in kanuka extracts with similar phytochemical profiles [12].

LC–MS analysis identified multiple phenolic compounds, including (−)-epigallocatechin, epicatechin 3′-O-glucuronide, and quercetin 3′-O-glucuronide, along with several additional polyphenols detected by MRM transitions. These results confirm the chemical complexity of *kanuka* foliage, in agreement with previous reports describing inter- and intraspecific variations in phenolic and terpenoid compositions among *Kunzea* species in New Zealand [10,12]. The LC-MS analysis showed the presence of diverse flavonoids, polyphenols and phenolic acids in kanuka leaf extract, suggesting possible synergistic interactions that may enhance the overall antioxidant efficacy of the extract.

The methanol extract, which contained the highest TPC and exhibited strong antioxidant activity, was subsequently evaluated for cytotoxicity against triple-negative breast cancer (TNBC) cell lines. The extract reduced cell viability in a concentration-dependent manner, with viability falling below 50% at dilutions lower than 1:1600. This outcome indicates that phenolic constituents in kanuka may exert bioactivity beyond antioxidation, potentially disrupting cancer cell metabolism or signaling pathways. However, the observed plateau in cytotoxicity at higher concentrations likely reflects the complex interplay between cytotoxic and antioxidant components within the crude extract. Similar biphasic dose–response behaviors have been observed in other plant-based systems containing multiple bioactive metabolites [10].

Overall, this study provides further evidence supporting the biochemical richness and therapeutic potential of kanuka. The superior extraction efficiency of methanol, combined with the extract’s antioxidant and cytotoxic properties, underscores its value as a natural source of multifunctional bioactive compounds. These findings are consistent with previous research demonstrating kanuka’s antioxidant, anti-inflammatory, and protective effects in cellular models [12,13]. Future work should aim to isolate and characterize the individual active constituents, investigate their molecular mechanisms of action, and explore their potential synergistic interactions with conventional chemotherapeutic agents to enhance efficacy while reducing toxicity.

## 5. Conclusions and Study Limitations

This study demonstrated that solvent polarity significantly influences the extraction efficiency, phytochemical composition, and biological activities of Kanuka (*Kunzea ericoides*) leaf extracts. The 50% methanol extract exhibited a higher total phenolic content and stronger antioxidant activity than the 70% acetone extract, attributable to methanol’s superior polarity and hydrogen-bonding capacity. LC–MS analysis confirmed the presence of multiple bioactive phenolics, including (−)-epigallocatechin, epicatechin 3′-O-glucuronide, and quercetin 3′-O-glucuronide, supporting the extract’s antioxidant potential. The methanol extract also displayed concentration-dependent cytotoxicity against triple-negative breast cancer (TNBC) cell lines, indicating that *kanuka* phenolics may possess complementary anticancer properties. Collectively, these results highlight *kanuka* leaves as a promising source of multifunctional phytochemicals with potential applications in antioxidant therapies, cancer research, and functional skincare formulations.

Despite these promising findings, several limitations should be acknowledged. The study employed crude solvent extracts, which contain complex mixtures of compounds that may exert synergistic or antagonistic effects; therefore, the precise contribution of individual phenolics remains unresolved. The solvent systems explored were limited to methanol and acetone; inclusion of other green or non-toxic solvents could provide further insight into optimal extraction strategies. Finally, mechanistic studies are required to elucidate the molecular pathways underlying the observed antioxidant and cytotoxic effects. Future work should focus on purification, structural elucidation, and mechanistic evaluation of key phenolic constituents, as well as exploring their potential synergistic interactions and therapeutic relevance in vivo.

## Figures and Tables

**Table 1:** Correlation between total phenolic content (TPC) and antioxidant activity measured by FRAP and CUPRAC assay in kanuka leaf extract prepared by 50% methanol and 70% acetone as solvents.

	Content	TPC	CUPRAC	FRAP
TPC	50% Me-OH	1	0.845	−0.727
	70% Acetone	1	−0.574	0.320
CUPRAC	50% Me-OH	0.845	1	−0.247
	70% Acetone	−0.574	1	−0.959
FRAP	50% Me-OH	−0.727	−0.247	1
	70% Acetone	0.320	−0.959	1

**Table 2:** List of potential polyphenols present in kanuka leaf extract based on TIC scan.

RT	M/Z	ID1	ID2	ID3	ID4	Signal
4.67	305	(-)-Epigallocatechin				Prominent
7.34	269	3′-Hydroxydaidzein	6,7,4′-Trihydroxyisoflavone	7,8,4′-Trihydroxyisoflavone		Weak
10.372	183	3-O-Methylgallic acid	4-O-Methylgallic acid			Weak
10.872	269	3′-Hydroxydaidzein	6,7,4′-Trihydroxyisoflavone	7,8,4′-Trihydroxyisoflavone	Genistein	Weak
11.357	337	5-p-Coumaroylquinic acid				Very weak
14.976	305	(-)-Epigallocatechin				Prominent
16.692	337	5-p-Coumaroylquinic acid				Very weak
19.304	479	3′-O-Methyl-(-)-epicatechin 7-O-glucuronide	4′-O-Methyl-(-)-epicatechin 3′-O-glucuronide	Paeoniflorin		Weak peak
19.863	463	Quercetin 3-O-glucoside				Prominent
20.408	466	Epicatechin 3′-O-glucuronide	Epicatechin 7-O-glucuronide			Prominent
20.945	477	Quercetin 3′-O-glucuronide	Quercetin 3-O-glucuronide	Quercetin 4′-O-glucuronide	Hesperetin 3′-O-glucuronide	Prominent
21.191	479	3′-O-Methyl-(-)-epicatechin 7-O-glucuronide	4′-O-Methyl-(-)-epicatechin 3′-O-glucuronide	Paeoniflorin		Weak peak
21.438	479	3′-O-Methyl-(-)-epicatechin 7-O-glucuronide	4′-O-Methyl-(-)-epicatechin 3′-O-glucuronide	Paeoniflorin		Weak peak
28.414	269	3′-Hydroxydaidzein	6,7,4′-Trihydroxyisoflavone	7,8,4′-Trihydroxyisoflavone	Genistein	Weak peak

RT-Retention time, *m*/*z*-Mass to charge ratio, ID 1, 2 and 3-Identify compounds using database.

## Data Availability

Data is contained within the article. The original data presented in the study is included in the article. For further inquiries, the corresponding author should be contacted.
